# Single nucleotide polymorphisms in the development of osteomyelitis and prosthetic joint infection: a narrative review

**DOI:** 10.3389/fimmu.2024.1444469

**Published:** 2024-09-05

**Authors:** Jia-Qi Zhou, Zi-Xian Liu, Hong-Fa Zhong, Guan-Qiao Liu, Ming-Cong Ding, Yu Zhang, Bin Yu, Nan Jiang

**Affiliations:** ^1^ Division of Orthopaedics & Traumatology, Department of Orthopaedics, Nanfang Hospital, Southern Medical University, Guangzhou, China; ^2^ Department of Orthopaedics, Guangdong Provincial People’s Hospital, Guangdong Academy of Medical Sciences, Southern Medical University, Guangzhou, China; ^3^ Guangdong Engineering Technology Research Center of Functional Repair of Bone Defects and Biomaterials, Guangdong Academy of Medical Sciences, Southern Medical University, Guangzhou, China; ^4^ The Second Hospital & Clinical Medical School, Lanzhou University, Lanzhou, China; ^5^ Department of Orthopedics, Lanzhou University Second Hospital, Lanzhou, China; ^6^ Department of Trauma Emergency Center, Ganzhou Hospital-Nanfang Hospital, Southern Medical University, Ganzhou, China

**Keywords:** osteomyelitis, prosthetic joint infection, single nucleotide polymorphisms, single nucle otide variations, narrative review

## Abstract

Currently, despite advancements in diagnostic and therapeutic modalities, osteomyelitis and prosthetic joint infection (PJI) continue to pose significant challenges for orthopaedic surgeons. These challenges are primarily attributed to the high degree of heterogeneity exhibited by these disorders, which are influenced by a combination of environmental and host factors. Recent research efforts have delved into the pathogenesis of osteomyelitis and PJI by investigating single nucleotide polymorphisms (SNPs). This review comprehensively summarizes the current evidence regarding the associations between SNPs and the predisposition to osteomyelitis and PJI across diverse populations. The findings suggest potential linkages between SNPs in genes such as *IL-1*, *IL-6*, *IFN-γ*, *TNF-α*, *VDR*, *tPA*, *CTSG*, *COX-2*, *MMP1*, *SLC11A1*, *Bax*, *NOS2*, and *NLRP3* with the development of osteomyelitis. Furthermore, SNPs in genes like *IL-1*, *IL-6*, *TNF-α*, *MBL*, *OPG*, *RANK*, and *GCSFR* are implicated in susceptibility to PJI. However, it is noted that most of these studies are single-center reports, lacking in-depth mechanistic research. To gain a more profound understanding of the roles played by various SNPs in the development of osteomyelitis and PJI, future multi-center studies and fundamental investigations are deemed necessary.

## 1 Introduction

Bone and joint infections, such as osteomyelitis and orthopedic implant-associated infections (1), have posed a persistent challenge in the field of orthopedics. Osteomyelitis, characterized by irregular bone neoformation and progressive bone deterioration (2, 3), remains a complex condition, despite the combined efforts of medical and surgical interventions. Alarmingly, even with adequate treatment, up to 30% of osteomyelitis cases may develop into chronic stage, resulting in substantial financial burden, morbidity, and mortality (4).

Total joint replacement stands as a highly effective treatment option for patients suffering from advanced osteoarthritis (5). Nevertheless, certain factors, including aseptic prosthesis loosening and prosthetic joint infection (PJI), can significantly influence the durability of artificial joints (6). Despite the routine employment of contemporary surgical techniques and antibacterial prophylaxis, some surgical patients continue to experience PJI (7). Therefore, a thorough comprehension of the underlying pathogenesis of bone and joint infections is paramount.

The pathogenesis of these infections is intricately linked to both environmental and host components. Notably, there is a growing body of evidence indicating that single nucleotide polymorphisms (SNPs) play a crucial role in the development of bone and joint infections (8, 9). SNPs refer to variations in a single nucleotide that arise at precise locations within the genome, possessing the potential to modulate an individual’s response to disease development (10). The comprehension of the significance of SNPs in the context of osteomyelitis and PJI is paramount, as these genetic alterations can influence the immune response, predisposition to infections, and disease progression (11, 12). The identification of specific SNPs correlated with these infections holds the potential to aid in the development of targeted preventive measures and individualized therapeutic approaches, ultimately enhancing patient outcomes (13). Current research has amassed increasing evidence suggesting that SNPs in various genes, including *IL-1*, *IL-6*, *IFN-γ*, *TNF-α*, *VDR*, *tPA*, *CTSG*, *COX-2*, *MMP1*, *SLC11A1*, *Bax*, *NOS2*, and *NLRP3*, may contribute to the development of osteomyelitis. Analogously, SNPs in the genes, such as *IL-1*, *IL-6*, *TNF-α*, *MBL*, *OPG*, *RANK*, and *GCSFR*, have been associated with susceptibility to PJI (**Table 1**). These genetic insights are fundamental in understanding the diversity of host responses to infections and in identifying patients who may be at a heightened risk. However, it is noteworthy that most studies conducted hitherto have been single-center reports, lacking in-depth mechanistic investigations, thus emphasizing the necessity for future multi-center studies and foundational research.

**Table 1:** SNPs involving in the development of bone and joint infections.

Authors	Population or ethnicity	Sample size (patients vs. healthy controls)	Genes	SNPs reported	Potential influences	Genotypes as risk or protective factors
Tsezou et al. (14)	Greek	81 vs. 110	*IL-1α*	rs1800587	Risk factor of osteomyelitis	CT+TT
Asensi et al. (15)	Spain	52 vs. 109	*IL-1α*	rs1800587	Risk factor of osteomyelitis	TT
Alves De Souza et al. (16)	Northeast Brazilian	39 vs. 114	*IL1RN*	rs2234663	Risk factor of post-traumatic osteomyelitis	2*/2*+L/*2
Jiang et al. (17)	China	189 vs. 200	*IL1RN*	rs4251961	Protective factor of post-traumatic osteomyelitis	C, CC+CT / CT
Jiang et al. (17)	China	189 vs. 200	*IL-1β*	rs16944	Risk factor of post-traumatic osteomyelitis	GG+AG / GG / AG
			*IL-1β*	rs1143627	Risk factor of post-traumatic osteomyelitis	TT+CT / TT
Yao et al. (18)	China	223 vs. 200	*IL-1β*	rs16944	Risk factor of extremity chronic osteomyelitis	GG+AG / AG
Asensi et al. (15)	Spain	52 vs. 109	*IL-1β*	rs1143634	Risk factor of osteomyelitis	TT
Osman et al. (19)	Saudi	52 vs. 103	*IL-1β*	rs16944	Protective factor of hematogenous osteomyelitis	G, GG, AA
Tsezou et al. (14)	Greek	81 vs. 110	*IL-4*	rs2243248	Risk factor of osteomyelitis	GT+TT
			*IL-4*	rs2243250	Risk factor of osteomyelitis	CT+TT
Osman et al. (20)	Saudi	52 vs. 103	*IL-4*	rs2070874	Risk factor of hematogenous osteomyelitis	C, CC
			*IL-4*	rs2070874	Protective factor of hematogenous osteomyelitis	CT
			*IL-4*	rs2243248	Risk factor of hematogenous osteomyelitis	GT
Jiang et al. (17)	China	189 vs. 200	*IL-6*	rs1800796	Risk factor of post-traumatic osteomyelitis	CC+CG / CC / CG
Tsezou et al. (14)	Greek	81 vs. 110	*IL-6*	rs1800795	Risk factor of osteomyelitis	GC+CC
Osman et al. (20)	Saudi	52 vs. 103	*IL-10*	rs1800871	Risk factor of hematogenous osteomyelitis	A
Osman et al. (20)	Saudi	52 vs. 103	*IL-12B*	rs3212227	Risk factor of hematogenous osteomyelitis	GG
Hou et al. (21)	China	233 vs. 200	*TNF-α*	rs1799964	Risk factor of extremity chronic osteomyelitis	T, TT
Zhao et al. (22)	China	189 vs. 200	*IFN-γ*	+874T/A	Risk factor of post-traumatic osteomyelitis	AT
Wang et al. (23)	China	189 vs. 220	*COX-2*	rs689466	Risk factor of post-traumatic osteomyelitis	GG
Osman et al. (19)	Saudi	52 vs. 103	*TLR2*	rs3804099	Risk factor of hematogenous osteomyelitis	C
			*TLR2*	rs3804099	Protective factor of hematogenous osteomyelitis	TT
Montes et al. (9)	Spain	80 vs. 155	*TLR4*	rs4986790	Risk factor of osteomyelitis	GG
				rs4986791	Risk factor of osteomyelitis	TT
Qu et al. (24)	China	306 vs. 368	*NLRP3*	rs10754558	Risk factor of post-traumatic osteomyelitis	CG
			*NLRP3*	rs7525979	Protective factor of post-traumatic osteomyelitis	TT
Pérez-Is et al. (25)	Spain	329 vs. 415	*CTSG*	rs45567233	Risk factor of osteomyelitis	G, AG
Ocaña et al. (26)	Spain	80 vs. 220	*Bax*	G (-248) A	Risk factor of osteomyelitis	A
Song et al. (27)	China	336 vs. 368	*NOS2*	rs2297514	Protective factor of post-traumatic osteomyelitis	CC
Zhao et al. (28)	China	336 vs. 368	*VDR*	rs7975232	Protective factor of fracture-related infection	AA
Zhao et al. (8)	China	398 vs. 368	*VDR*	rs7975232	Protective factor of osteomyelitis	AA
			*VDR*	rs1544410	Protective factor of osteomyelitis	CT
Jiang et al. (29)	China	223 vs. 200	*VDR TaqI*	rs731236	Risk factor of extremity chronic osteomyelitis	C, CC+CT
			*VDR FokI*	rs2228570	Risk factor of extremity chronic osteomyelitis	C, CC+CT / CC
Valle-Garay et al. (30)	Spain	261 vs. 299	*tPA*	rs4646972	Risk factor of bacterial osteomyelitis	I, I/I
Montes et al. (2)	Spain	118 vs. 300	*MMP1*	rs1799750	Risk factor of osteomyelitis	2G, 2G/2G
Kong et al. (31)	China	80 vs. 81	*MMP1*	rs1799750	Risk factor of osteomyelitis	2G / 2G/2G
			*MMP1*	rs1144393	Risk factor of osteomyelitis	G, GG
Jiang et al. (32)	China	336 vs. 368	*SLC11A1*	rs17235409	Risk factor of post-traumatic osteomyelitis	AG
			*SLC11A1*	rs3731865	Protective factor of post-traumatic osteomyelitis	CC+CG

SNPs, Single Nucleotide Polymorphisms; IL1RN, Interleukin-1 Receptor Antagonist Cytokine Gene; TLR, Toll-like Receptor; tPA, Tissue Plasminogen Activator; IFN-γ, Interferon gamma; CTSG, Cathepsin G; NOS2, Nitric oxide synthase 2; COX-2, Cyclooxygenase-2; VDR, Vitamin D Receptor; SLC11A1, Solute carrier family 11 member 1; MMP1, Matrix Metalloproteases 1; IL, Interleukin; Bax, Bcl-2-associated X; TNF-α, Tumor necrosis factor-alpha; NLRP3, NOD-like receptor thermal protein domain associated protein 3.

This comprehensive review encapsulates the current evidence pertaining to the associations between SNPs of diverse genes and the predisposition to osteomyelitis and PJI across various populations. It underscores the significant potential of genetic research in augmenting our comprehension and management strategies for bone and joint infections.

## 2 SNPs involved in osteomyelitis

### 2.1 Interleukin-1

Interleukin-1 (IL-1), serving as a pivotal regulator of innate immunity and inflammation, manifests in two distinct forms: IL-1α and IL-1β (16, 18). Notably, *IL-1β*, the gene encoding the IL-1β cytokine, exhibits a high degree of polymorphism. Furthermore, IL-1 receptor antagonist (IL-1Ra), a naturally occurring competitive inhibitor, binds to the same receptor as IL-1α and IL-1β, yet it does not elicit an inflammatory response (33). Recent investigations have revealed a close association between IL-1 and bone homeostasis as well as bone-related disorders (34).

Extensive research has focused on elucidating the potential associations between the SNPs of *IL-1* and the development of osteomyelitis. In a case-control study conducted in 2008 by Tsezou et al. (14), an examination was made of the relationship between *IL-1α* SNPs and chronic osteomyelitis in a Greek cohort. The study encompassed 81 patients diagnosed with osteomyelitis and 110 healthy controls. The results revealed a nearly sevenfold increase in the risk of osteomyelitis among the TT individuals carrying the *IL-1* -889-C/T polymorphism. Comparable findings were observed in a study conducted by Asensi et al. (15) on a Spanish population. Additionally, studies have shown an elevation in IL-1 levels in the serum of rodents suffering from experimental post-traumatic osteomyelitis (PTOM) (35). Consequently, it is plausible to posit that the *IL-1* -889-C/T mutation represents a significant risk factor for the development of osteomyelitis. However, given that both aforementioned studies were conducted at a single center and involved a relatively small number of participants, it is imperative to conduct further multicenter studies with a larger sample size to validate these findings.

In 2003, Asensi et al. (15) documented a correlation between the *IL-1β* gene polymorphism (rs1143634) and the likelihood of developing osteomyelitis in a Spanish cohort. Their findings indicated that individuals with TT genotype exhibited a heightened risk of osteomyelitis compared to those with CT and CC genotypes. A subsequent case-control study conducted by Yao et al. (18) in 2015 revealed a potential association between the *IL-1β* gene rs16944 and an increased predisposition for chronic osteomyelitis among an Chinese Han population, specifically those with AG genotype. Furthermore, they observed a potential link between the *IL-1* gene polymorphism (rs1143627) and an elevated risk of chronic osteomyelitis across dominant, homozygote, and heterozygous models, albeit statistical significance in the comparison between patients and healthy controls was not achieved. Of particular note, Osman et al. (19) conducted a case-control study encompassing 52 patients with hematogenous osteomyelitis and 103 healthy controls, and discovered that the G allele of rs16944 was significantly associated with a diminished risk of hematogenous osteomyelitis in a Saudi population. A recent case-control study (17) reported a heightened propensity for PTOM among individuals with the rs16944 genotypes AG and GG, rs1143627 genotype TT, and rs1800796 genotypes CG and GG. However, their findings regarding the influence of *IL-1* gene polymorphism (rs16944) on osteomyelitis susceptibility are inconsistent. This suggests the need for a more nuanced examination of different osteomyelitis subtypes and an independent exploration of the relationships between *IL-1β* gene polymorphisms and these varying types of osteomyelitis. Additionally, population heterogeneity may serve as a contributing factor.

The presence of variable numbers of tandem repeats (VNTR, 86-bp repeats) within intron 2 of the *IL-1 receptor antagonist cytokine* (*IL-1 RN*) gene constitutes a significant genetic polymorphism. Alves De Souza et al. (16) reported a more than twofold increased risk of osteomyelitis in a model dominated by the short allele (IL1RN*2) with two repetitive sequences. Furthermore, their study revealed that individuals harboring the IL1RN*2/*2 + *IL1B-*511T/T polymorphism genotype were all afflicted with osteomyelitis. The infrequent association and separation of the two polymorphism alleles (*IL1RN VNTR* and *IL1B*-*511C > T*) by 280kb (36) suggests a causal relationship rather than a mere linkage disequilibrium. A study conducted by Jiang et al. (17) in 2020 examined the potential correlation between the *IL-1RN* SNP rs4251961 and susceptibility to PTOM in a Chinese Han population. The C allele of rs4251961 emerged as an anti-osteomyelitis factor among the 389 participants.

The polymorphisms in the *IL-1* gene, by modulating the levels of IL-1 receptor antagonist (IL-1 Ra), IL-1α, and IL-1β at inflammatory sites, significantly influence the inflammatory response, including its initiation, persistence, and resolution (33). However, the precise impact of these polymorphisms on the cytokine levels and the underlying mechanisms contributing to osteomyelitis development remain unclear and warrant further exploration.

### 2.2 Interleukin-4 and Interleukin-6

In the evolution of chronic osteomyelitis, a crucial aspect is the appearance of necrotic bone fragments (37). It has been definitively established that IL-4 and IL-6 play a direct role in bone resorption and the regulation of osteoclast activity in chronic osteomyelitis (14). Moreover, patients afflicted with osteomyelitis have exhibited elevated levels of IL-4 and IL-6 (38). Recent studies have uncovered a correlation between the development of osteomyelitis and specific SNPs in *IL-4* (rs2243248, rs2243250, and rs2070874) as well as SNPs in *IL-6* (rs1800795, rs1800796).

In a case-control study conducted in 2008, Tsezou et al. (14) observed a significant association between two polymorphisms of the *IL-4* gene (rs2243248 and rs2243250) and elevated risks of osteomyelitis. Specifically, individuals carrying the T allele exhibited a heightened risk of developing osteomyelitis. Additionally, the study found that individuals with the homozygote for the C allele of *IL-6* rs1800795 C allele were at an increased risk of developing osteomyelitis. In 2015, Osman et al. (20) reported that in an Saudi population, the *IL-4* rs2243248 genotype GT was associated with a greater likelihood to develop hematogenous osteomyelitis. They further discovered that the heterozygous genotype (CT) of *IL-4* rs2070874 exhibited protective effects compared to the CC and TT genotypes, while the C allele was identified as a risk factor for hematogenous osteomyelitis. Jiang et al. (17) observed a correlation between the *IL-6* gene rs1800796 and an augmented vulnerability to PTOM, where individuals with CG and CC genotypes had a higher propensity for developing this condition. However, it is noteworthy that these findings are derived from single-center studies with limited sample sizes, thus necessitating further confirmation through multicenter studies encompassing larger sample sizes in the future. While numerous studies have uncovered associations between *IL-4* and *IL-6* SNPs and various inflammatory diseases, the underlying mechanisms remain unclear.

### 2.3 Interleukin-10

IL-10 was initially identified for its capability to restrain the activation and effector functions of immune cells (e.g. T cells, monocytes, and macrophages) (39). Beyond its traditional function of limiting and ultimately terminating inflammatory responses, IL-10 has been identified as a pivotal player in the differentiation and functioning of T regulatory cells (39). Recent investigations have uncovered a correlation between *IL-10* SNPs and a spectrum of inflammatory disorders, such as sepsis (40), osteomyelitis (20), and pancreatitis (41).

Osman et al. (20) conducted a rigorous case-control study in 2015 to investigate potential association between *IL-10* SNP rs1800871 polymorphism and the predisposition to hematogenous osteomyelitis among a Saudi population. The analysis of 155 subjects revealed that the presence of the A allele of the *IL-10* rs1800871 variant constituted a significant risk factor for the development of hematogenous osteomyelitis. Furthermore, a study by Hofmann et al. (42) demonstrated that individuals afflicted with chronic recurrent multifocal osteomyelitis exhibited diminished IL-10 expression, which subsequently triggered the NLRP3 inflammasome and contributed to inflammatory bone loss. These cumulative findings suggest that *IL-10* SNP rs1800871, through its ability to modulate IL-10 levels and further influence the activation of the NLRP3 inflammasome, may exert an influence on the progression of osteomyelitis. However, further in-depth mechanistic studies are imperative to elucidate these complex interactions.

### 2.4 Interleukin-12

Interleukin-12 (IL-12) is a cytokine that promotes inflammation, produced primarily by non-T cells, especially antigen-presenting cells integral to the T-helper 1 system (43). IL-12 exerts its effects primarily on T cells and natural killer cells, exhibiting numerous biological effects. The *IL-12B* gene is responsible for encoding a component of IL-12. Recent investigations have identified a linkage between variations in the *IL-12B* gene and various infectious diseases, including chronic hepatitis B (43), Q fever caused by Coxsackie burnetii (44), and chronic osteomyelitis (20). Among these variations, the SNP locus rs3212227 has garnered significant attention in research.

In a case-control study conducted in 2015, Osman et al. (20) conducted an analysis to examine the potential association between SNP rs1800871 in the *IL-12B* gene and susceptibility to hematogenous osteomyelitis among a Saudi population. The findings revealed that individuals possessing GG genotype exhibited a heightened risk of developing osteomyelitis in comparison to those with GT and TT genotypes. While Osman et al. did not delve deeper into the underlying mechanisms, research has demonstrated that endogenous IL-12 production or the exogenous administration of recombinant IL-12 enhances the body’s immunity against a diverse array of cellular pathogens (45). Furthermore, a substantial component of this defense mechanism appears to be influenced by IL-12, which prompts T lymphocytes and natural killer cells to produce interferon (46). This increased interferon stimulates macrophages and T lymphocytes, thereby enabling them to more effectively eliminate pathogens from invaded cells (47). *S. aureus*, the most prevalent pathogen in all forms of osteomyelitis, has the ability to colonize the bone matrix and be internalized and retained by osteoblasts, potentially contributing to the development of chronic infection (45). Consequently, polymorphisms in the *IL-12B* gene may prevent the development of osteomyelitis by modulating serum IL-12 levels and the IL-12/interferon pathway. However, further research is imperative as the current understanding of the relationship between osteomyelitis and *IL-12B* polymorphisms remains limited.

### 2.5 Tumor necrosis factor-alpha

Tumor necrosis factor (TNF), a crucial proinflammatory cytokine primarily secreted by macrophages, plays a pivotal role in the progression of numerous infectious disorders. Alterations in the DNA sequence within regulatory regions can potentially alter transcriptional regulation, thereby affecting TNF levels in the bloodstream and augmenting vulnerability to various infectious diseases (48). Recent investigations have indicated that genetic variants in the promoter region of the *TNF-α* gene, specifically SNPs, may modulate the secretion of TNF-α (49). Among the most extensively studied *TNF-α* gene polymorphism is rs1799964.

In a case-control study of 433 subjects (233 patients with extremity chronic osteomyelitis and 200 healthy controls), Hou et al. (21) reported that the frequency of the T allele in rs1799964 was significantly higher in case group subjects than that in the control subjects, indicating that having the rs1799964 T allele might be a potential risk factor. Additionally, they determined that there was a statistical connection between the quantity of leukocytes in patients and the *TNF-α* gene polymorphism rs1799964. The potential link between *TNF-α* SNP and leukocyte counts needs to be further investigated. Ma et al. (50) found that during *S. aureus* infection, elevated TNF-α enhanced the expression of endogenous miR-129-5p, inhibiting endothelial nitric oxide synthase (eNOS), proving the role of TNF-α/miR-129-5p/eNOS in the pathogenesis of osteomyelitis. There was no evidence to support either the leukocyte/inflammatory factor route or the TNF-α/miR-129-5p/eNOS pathway as the mechanism by which *TNF-α* gene polymorphisms caused osteomyelitis. Moreover, considering that the study by Hou et al. was a single-center study with a limited number of participants, there is a need for future multicenter studies with larger sample sizes.

### 2.6 Interferon gamma

Interferon gamma (IFN-γ) serves as a pivotal immunomodulatory factor, derived from T cells and natural killer cells (NK cells), in the context of immune responses (51). IFN-γ plays a crucial role in the anti-infective immune response to infection, primarily through augmenting antigen presentation and phagocytosis in macrophages (52). Additionally, IFN-γ has been employed in the therapeutic management of certain chronic and inflammatory disorders (53, 54), thereby indicating its potential significance in the immune regulation of osteomyelitis (55). However, it is noteworthy that genetic polymorphisms can lead to variations in IFN-γ production levels and immune responsiveness, ultimately influencing an individual’s resilience to pathogens.

Zhao et al. (22) conducted a study which revealed that the A allele in the *IFN-γ* +874T/A polymorphism appears to heighten an individual’s predisposition towards PTOM. Specifically, the presence of the A allele, particularly in AT heterozygotes, was observed to significantly increase the likelihood of osteomyelitis development compared to the TT heterozygote. Furthermore, the research suggests that genetic variations in *IFN-γ* +874T/A may modulate an individual’s vulnerability to PTOM by influencing serum IFN-γ concentrations. Specifically, the A allele is postulated to be associated with reduced IFN-γ levels, potentially impairing the ability to mount an effective antimicrobial response and subsequently elevating the risk of PTOM. In essence, the *IFN-γ* +874T/A gene polymorphisms may serve as a potential marker for future genetic screening and personalized therapeutic approaches in the context of PTOM.

### 2.7 Toll-like receptors

Toll-like receptors (TLRs) function as pattern recognition receptors that identify pathogen-associated molecular patterns and subsequently trigger the immune system and host defense-stimulating activities (56). TLRs influenced the release of pro-inflammatory cytokines by interacting with pathogen-associated molecular patterns (19). However, excessive release of pro-inflammatory factors inhibited tissue regeneration. Recent research has demonstrated that *TLR* SNPs play a role in the development of osteomyelitis.

In a case-control study published in 2016, Osman et al. (19) examined the relationship between the *TLR2* gene polymorphism rs3804099 and hematogenous osteomyelitis susceptibility in a Saudi population. They noted that people with C allele had a higher risk to develop osteomyelitis. It was reported by Montes et al. (9) that osteomyelitis was linked to the *TLR4* polymorphism rs4986790’s GG genotype, regardless of the frequency of the G allele. This finding would suggest that the mutation has a dosage effect and needs both alleles to have the full pathogenic effect. Similar results were found in the *TLR4* polymorphism rs4986791. It has been reported that aureus peptidoglycan promotes osteoclast formation through TLR2-mediated activation of the nuclear factor kappa-B (NF-κB)/nuclear factor of activated T cells 1 (NFATc1) signaling pathway (57), causing bone destruction and the formation of osteomyelitis. The association between *TLR2* polymorphisms and vulnerability to osteomyelitis and this signaling pathway, however, has not been proven. Neutrophils typically have a short half-life and pass away by apoptosis. Further research by Montes et al. (9) revealed that individuals with the G allele of the *TLR4* (rs4986790) polymorphism have considerably lower neutrophil apoptosis and reactivity to lipopolysaccharide, which may help explain the persistent nature of bone infections. The specific mechanism influencing the osteomyelitis pathogenesis requires to be identified to fully understand the association between *TLR* polymorphisms and osteomyelitis susceptibility.

### 2.8 Vitamin D receptor

Vitamin D receptor (VDR) is encoded by the *VDR* gene located on chromosome 12. VDR participates in several biological processes, including the modulation of immune response and bone metabolism (28, 29). High polymorphism existed in the *VDR* gene. Studies already conducted have shown that *VDR* gene variations are linked to an increased risk of contracting several inflammatory disorders, including tuberculosis (58), chronic periodontitis (59), and leprosy (60). The *VDR* gene variations *TaqI* (rs731236), *BsmI* (rs1544410), *ApaI* (rs7975232), and *FokI* (rs2228570) have been the subjects of the most studies.

In a 2016 case-control study, Jiang et al. (29) reported the association between the *VDR* gene polymorphisms rs731236 and rs2228570 and susceptibilities to chronic osteomyelitis in a Chinese population and found that the frequencies of the C allele in both rs731236 and rs2228570 were significantly higher in subjects in the case group than in those in the control group. Furthermore, the homozygote model revealed a substantial association between the likelihood of acquiring chronic osteomyelitis and the gene polymorphisms rs731236 and rs2228570. Therefore, people having the C allele in the *VDR* gene polymorphisms rs731236 and rs2228570 were more likely to develop osteomyelitis. By using recessive and homozygote models, Zhao et al. (28) observed a significant connection between the *VDR* gene SNP rs7975232 and a risk of developing fracture-related infections, suggesting that individuals with the AA genotype at this location are less vulnerable to these infection. Zhao et al. (8) similarly found that rs7975232 was associated with susceptibility to osteomyelitis, with the AA genotype as a protective factor. In addition to this, the heterozygote model showed a significant correlation between rs1544410 and susceptibility to osteomyelitis, suggesting that the CT genotype may have a protective effect against osteomyelitis. Through *in vivo* and *in vitro* experiments, Zhao et al. (8) also showed that the protective effect of vitamin D against osteomyelitis may be attained in part by preventing macrophage death by inhibiting excessive reactive oxygen species (ROS) production by the VDR-Bmi1 signaling pathway.

### 2.9 Tissue plasminogen activator

Tissue plasminogen activator (tPA), encoded by the human gene *t-PA* located on chromosome 8p11.21, is a key protease of the fibrinolytic system (61). The *tPA* Alu insertion/deletion [I/D] is a 311 bp sequence inserted/deleted in the 8th intron of the *tPA* gene (30). Studies that are now available have demonstrated definite associations between *tPA* polymorphism and numerous disorders, such as stroke (61) and bacterial infections (62).

In a 2013 case-control study, Valle-Garay et al. (30) reported that the *tPA* polymorphism (rs4646972) was associated with an increased susceptibility to bacterial osteomyelitis in a Spanish population, with the I allele as a risk factor. They also noted that patients with osteomyelitis had a considerably higher prevalence of the I/I genotype compared to controls. The release rate of tPA was considerably greater in people with the I/I genotype than in those with other genotypes, according to Jern et al.’s (63) study of subjects with various genotypes of the *tPA* polymorphism (rs4646972). This accelerated rate of tPA release may be involved in the development of osteomyelitis through an *in vivo* process. However, further studies are needed.

### 2.10 Cathepsin G

Cathepsin G (CTSG), encoded by the human gene *CTSG* gene located on chromosome 14q11.2, is a 26-kDa serine protease (25). CTSG was hypothesized to be connected to tissue remodeling at locations of tissue damage, neutrophil response to different pathogens, and blood coagulation (64). In a 2019 study, Pérez-Is et al. (25) investigated the relationship between the *N125S* polymorphism of the *CTSG* gene and the risk of developing osteomyelitis, and the results suggested that the G allele and the AG genotype are risk factors for the development of osteomyelitis. In individuals with the G allele, they also discovered a substantial rise in serum CTSG activity and lactoferrin levels. The *CTSG* gene *N125S* polymorphism may therefore increase osteomyelitis susceptibility by increasing lactoferrin and *CTSG* activity. The precise molecular mechanism is still unclear, though. Pyroptosis, a process of inflammatory cell death, can trigger a potent inflammatory response to protect the host from microbial infection (65). A protein produced by Mycobacterium tuberculosis known as *Rv3364c* has been discovered to bind to host CTSG and prevent pyroptosis in infected macrophages, allowing bound mycobacteria to survive in macrophages (66). It is unclear if osteomyelitis development is governed by analogous mechanisms. To prove this, more research is required.

### 2.11 Cyclooxygenase-2

Cyclooxygenase-2 (COX-2) is encoded by the *COX-2* gene located on chromosome 1q25.2-25.3 with a transcript of 4.5 kb (67). In the presence of COX, free arachidonic acid is converted to prostaglandins, regulating the inflammatory response process (68). *COX-2* polymorphisms have been reported to be associated with a variety of inflammatory diseases, such as inflammatory bowel disease (69), periodontitis (70), and asthma (71). Wang et al. (23) investigated potential associations of *COX-2* gene polymorphisms with susceptibility to osteomyelitis in a Chinese population. Results from 409 subjects (189 patients and 220 controls) showed a significant association between rs689466 and PTOM, with GG genotype as a risk factor. They also compared whether there were statistical differences in the levels of the six cytokines among the people with different genotypes to clarify the potential mechanisms of this polymorphism in the pathogenesis of osteomyelitis. But there were no statistically significant differences. Through an *in vivo* research, Johansen et al. (72) reported that COX-2-mediated prostaglandins play a role in early osteomyelitis bone resorption. However, the underlying mechanisms of *COX-2* polymorphisms in the pathogenesis of osteomyelitis remain unclear.

### 2.12 Nitric oxide synthase 2

Nitric oxide synthase 2 (NOS2), also known as inducible nitric oxide synthase (iNOS), is one of the three key enzymes in the production of nitric oxide (NO) in the human body (73). It has been shown that in chronic inflammatory diseases such as rheumatoid arthritis (74), asthma (75), and inflammatory bowel disease (76), polymorphisms in the *NOS2* gene may affect its expressions and activities, thereby influencing an individual’s susceptibility to inflammatory diseases (77). The study by Song et al. (27) included 336 patients with PTOM and 368 healthy controls. The rs2297514 and rs2248814 loci in the *NOS2* gene of the participants were genotyped by SNaPshot genotyping methods. It was found that the frequency of the C allele at the rs2297514 locus was significantly lower in the group of PTOM patients than that in the group of healthy controls (48.7% vs. 54.5%), suggesting that the C allele may have a protective role in reducing the susceptibility to PTOM. In addition, the median level of CRP was significantly lower in PTOM patients with the CC genotype than in those with the TT genotype (4.1 mg/L vs. 8.9 mg/L, *P* = 0.027), suggesting that the CC genotype may exert its protective effect by influencing the severity of the inflammatory response. However, no significant association was found between rs2248814 and PTOM susceptibility. This finding provides further evidence of potential links between polymorphisms in the *NOS2* gene and the pathogenesis of PTOM and may contribute to the development of future preventive and therapeutic strategies for such diseases.

### 2.13 Matrix metalloproteases 1

Matrix metalloproteases (MMPs) are zinc-dependent protein hydrolases that break down different extracellular matrix protein components and are crucial for both healthy physiological processes and pathological conditions (31, 78). One of the crucial MMPs, MMP1, is capable of dissolving type I collagen fibers (79). The emergence of chronic inflammation is strongly correlated with the *MMP1* gene SNP (80). In a case-control study in 2010, Montes et al. (2) reported that the *MMP1* polymorphism (rs1799750) was associated with an increased susceptibility to osteomyelitis in a Spanish population, with the 2G allele as a risk factor. The study by Kong et al. (31) produced similar outcomes. Along with rs1799750, Kong et al. (31) additionally observed that the G allele of the *MMP1* polymorphism rs1144393 increased the incidence of osteomyelitis. The serum of osteomyelitis patients included high levels of inflammatory mediators such TNF-α and IL-1, which mediated enhanced MMP expression (2). In cells with the 2G allele (rs1799750), Cao et al. (80) discovered changes in MMP1 protein and transcript levels. However, no additional research into the underlying molecular pathways has been done. Furthermore, research on the *MMP1* rs1144393 polymorphism’s pathogenic mechanism is scarce.

### 2.14 Bcl-2-associated X protein

Bcl-2-associated X protein (Bax), encoded by the *BAX* gene of the *BCL-2* gene family, is the predominant apoptotic gene in humans and functions by forming a heterodimer with Bcl-2 protein (81). A critical element in regulating the potency of the inhibitory impact on apoptosis is the ratio between the Bax*/*Bcl-2 proteins (82). The expression and function of the *Bax* gene are impacted by mutations in its coding area and promoter (26). In a case-control study in 2007, Ocaña et al. (26) investigated the association between the *Bax* polymorphism rs4645878 and chronic osteomyelitis in a Spanish population. A total of 80 patients with osteomyelitis as well as 220 healthy controls were included. The findings demonstrated that individuals with the A allele had a greater incidence of osteomyelitis occurence, lower expression of the *Bax* protein level, and a longer median survival time for peripheral neutrophils. More research is required to better understand how this polymorphism’s anti-apoptotic impact influences susceptibility to osteomyelitis.

### 2.15 NOD-like receptor thermal protein domain associated protein 3

NOD-like receptor thermal protein domain associated protein 3 (NLRP3) is an important pattern recognition receptor in the cytoplasm that, together with apoptosis-associated speck-like protein containing a CARD (ASC) and pro-cysteinyl aspartate specific proteinase-1 (pro-caspase-1), forms the NLRP3 inflammatory vesicle (83). The NLRP3 protein is encoded by a gene with nine exons that is found on chromosome 1q44 (24). Pro-inflammatory cytokines mature and pyroptosis was induced when NLRP3 inflammatory vesicles were activated (83). However, excessive activation of NLRP3 activation also promoted the development of a number of inflammatory disorders, including type 2 diabetes, inflammatory bowel disease, and gouty arthritis (84, 85). In a case-control study conducted in 2023, Qu et al. (24) discovered that the *NLRP3* gene polymorphisms rs10754558 and rs7525979 may be linked to PTOM susceptibility in an Chinese Han population, with the CG genotype (rs10754558) population possibly being at high risk and the TT genotype (rs7525979) population being protective. Although the pathogenic mechanism of the *NLRP3* polymorphism (rs10754558) has not been further explored by Qu et al., Zhang et al. (86) found that the polymorphism rs10754558 may be involved in regulating the immune and inflammatory response in patients with primary gouty arthritis by affecting the expression of NLRP3 inflammasome components. Whether the NLRP3/IL-1β signaling pathway is involved in susceptibility to osteomyelitis due to *NLRP3* polymorphism (rs10754558) is unclear. In a study by von Herrmann et al. (87), it was reported that the *NLRP3* polymorphism rs7525979 was connected to a considerably decreased risk of Parkinson’s disease development. They also showed that the polymorphism rs7525979 influences the translation efficiency of the NLRP3 protein, which in turn affects how stable, ubiquitinated, and soluble it is. Further research is necessary to determine whether comparable pathways are present in models of osteomyelitis.

### 2.16 Solute carrier family 11 member 1

*Solute Carrier Family 11 Member 1 (SLC11A1)*, is a gene that encodes a transmembrane protein that is expressed in macroscopic immune cells such as macrophages and plays an important role in the transport of iron and other metal ions (88). Polymorphisms in the *SLC11A1* gene may affect the function of this protein and thus the susceptibility to some diseases (89–92). The study by Jiang et al. (32) included 336 PTOM patients and 368 healthy controls. Participants were genotyped to assess the prevalence of the rs17235409 and rs3731865. Results indicated that rs17235409 was associated with an increased risk of developing PTOM. Specifically, the AG genotype of rs17235409 was found to increase risk, and patients with the AG genotype had relatively higher levels of inflammatory biomarkers than those with the AA and GG genotypes, particularly white blood cell counts and C-reactive protein levels. This suggests that this genotype may be a risk factor of PTOM development. Whereas rs3731865 was found to possibly reduce the risk of PTOM, although the results were not statistically significant. In conclusion, this study demonstrates a potential link between specific genetic variants in the *SLC11A1* gene and susceptibilities to PTOM, emphasizing the importance of genetic factors in the development of this disease. However, more research is needed to fully understand these mechanisms and confirm these findings, especially for rs3731865.

## 3 SNPs involved in PJI development

### 3.1 IL and TNF-α

IL and TNF-α are pivotal inflammatory mediators that play a fundamental role in the underlying mechanisms of aseptic prosthesis loosening (APL)/aseptic failure of arthroplasty, as well as in the pathogenesis of PJI (93). IL-1, IL-6 and TNF-α are closely related to osteoclastogenesis and thus play an important role in bone remodeling (94, 95). Levels of IL-1, IL-6, and TNF-α were observed to be elevated in individuals who have experienced arthroplasty failure (96). Furthermore, cytokines associated with the Th-17 immune response, which are instrumental in providing immune protection against microorganisms, are also potential genetic candidates that may confer an increased susceptibility to PJI (97).

In a 2012 case-control study, Stahelova et al. (98) investigated the relationships between genetic polymorphisms of *IL-1β*, *IL-6*, and *TNF-α* and susceptibilities to PJI, and enrolled 471 subjects (89 PJI vs. 214 APL vs. 168 healthy controls). They found that the *IL-1β* gene T allele of polymorphism rs16944 was a risk factor for PJI development, while there were no significant associations between *IL-6* (-174 G/C, A/G nt565) or *TNF-α* (rs1800629, rs361525) SNPs and PJI development. Similarly, Malik et al. (99) failed to find definite link between *IL-6-174* SNP and PJI occurrence. However, in a prospective study, Erdemli et al. (100) observed that the frequency of *IL-6*-174 *C* allele among the PJI patients was significantly higher than that among the APL patients.Meanwhile, they found that the G allele of the *TNF-α* polymorphism rs361525, IL1RN*2/*2 and IL1RN*1/*2 genotypes were associated with increased susceptibilities to PJI. Racial heterogeneity could potentially be a contributing factor to this discrepancy. Furthermore, the research conducted by Erdemli et al. was structured as a prospective investigation and did not incorporate a comparison group of healthy individuals. Furthermore, López-Anglada et al. (93) investigated potential associations between *IL-1β* SNP rs1143634 and susceptibilities to PJI in a Spanish population. Based on an analysis of 117 PJI patients, 77 APL patients, and 145 healthy controls, the findings revealed that, in comparison to the healthy controls, the presence of the *IL-1β* SNP rs1143634 T allele and the TT genotype were identified as risk factors for APL. Conversely, no such associations were observed among the PJI patients. Additionally, in line with the findings of Stahelova et al. (98), López-Anglada et al. (93) reported that no positive correlation was established between rs1800629 (TNF-α gene) and the development of PJI. Navratilova et al. (101) indicated that, despite observing an increased Th-17 immune response among individuals with PJI, no significant positive correlations were established between cytokine polymorphisms associated with Th-17 immunological reactivity and the susceptibility to PJI.

### 3.2 Mannose-binding lectin

Mannose-binding lectin (MBL), a serum protein derived from the liver, is intricately involved in the activation process of the complement pathway. Additionally, it elicits the activation of macrophages through the mediation of the C1q receptor. Consequently, MBL occupies a pivotal position in innate immunity, underscoring its critical role in the body’s natural defense mechanisms (102). Siassi et al. (103) observed that diminished levels of MBL may elevate the potential for postoperative infection, irrespective of whether they display an immediate reaction to surgical trauma. Furthermore, there exists a strong correlation between reduced MBL levels and SNPs within the *MBL2* gene, which is located on chromosome 10 (104). Clinical research has demonstrated that these SNPs are linked to increased risks of developing inflammatory conditions, such systemic lupus erythematosus (105) and arthritis (106). Malik et al. (102) conducted a rigorous investigation into the correlations between *MBL* SNPs located at the promoter position -500 G/C (MBL-550 G/C) and codon 54 G/A (MBL-54 G/A) and their potential impacts on the susceptibilities to PJI in a cohort from the UK. Their findings indicated that the C allele and the CC genotype of the -550 SNP, as well as the -54 GG genotype, serve as risk factors for the development of PJI. Similarly, Navratilova et al. (6) indicated that there were significant increases in the frequencies of the L allele and LL genotype of the MBL-550 SNP among the PJI patients, as compared to the controls and the APL patients. Furthermore, it was observed that individuals carrying the L allele exhibited lower serum MBL concentrations than those who did not carry the allele. This finding suggests that such a genetic variant may contribute to an elevated risk of PJI, potentially through the regulation of serological MBL levels. However, it is unfortunate to note that no subsequent studies were undertaken to elucidate the underlying molecular mechanisms linking low MBL levels to the occurrence of PJI.

### 3.3 Other genes related to PJI

Aside from *IL*, *TNF-α*, and *MBL*, polymorphisms in certain genes, including *TLRs*, *osteoprotegerin* (*OPG*), *receptor activator of NF-kappaB* (*RANK*), *VDR*, and *granulocyte colony stimulating factor receptor* (*GCSFR*), have been examined to determine their potential correlations with PJI development. Given the scarcity of research in this area and the fact that some of these genes have already been previously investigated, we shall delve into their discussions collectively in this context.

Despite TLRs’ pivotal role in innate immunity, the research conducted by Mrazek et al. (107) indicated that the presence of multiple genetic polymorphisms within TLRs did not amplify the predisposition to PJI. Interestingly, El-Helou and colleagues (108), through rigorous *in vitro* experimentation, observed that the TLR2 R753Q SNP effectively suppressed the cellular response triggered by *S. aureus* peptidoglycan. Furthermore, osteoclastogenesis exhibits a profound link with OPG and RANK, and it is noteworthy that bacterial infections may contribute to bone loss in the context of periprosthetic prostheses for total joint arthroplasty by initiating a series of reactions mediated by the OPG/RANK/RANKL pathway (109). In a case-control study conducted by Malik et al. (5), a significant association was observed between the A allele and AA genotype of the *OPG* SNP rs3102735, as well as the T allele and TT genotype of the *RANK* SNP at position 575 in exon 6 (*RANK* + 575 C/T), with APL. Additionally, the study revealed that, when compared to the control group, the A allele and AA genotype of the *OPG* SNP rs3102735 served as risk factors for the development of PJI.

The SNPs in the promoter region of the *OPG* gene can affect the expression levels of OPG proteins by modulating transcription, whereas the SNP in the *RANK* gene may alter the functionality of the resulting protein product. Malik et al. (99) observed that, when compared to controls, the presence of the T allele and the TT genotype of VDR Taqman Test (VDR-T) assay reagents intensified osteolysis caused by deep infections. Granulocyte Colony Stimulating Factor (GCSF), an inflammatory cytokine, binds to the GCSF receptor (GCSFR), thereby modulating inflammatory processes. Erdemli et al. (100) noted a significant association between the CT genotype of GCSFR and PJI. Nevertheless, research on these genetic polymorphisms remains limited in elucidating the precise relationship between them and the development of PJI. Consequently, there is a need for additional multicenter studies involving larger sample sizes.

## 4 Potential clinical applications

### 4.1 Prediction and screening

Detecting SNPs associated with osteomyelitis and PJI allows for an accurate assessment of risk for individuals, enabling earlier intervention and the implementation of preventive measures for high-risk populations (24). In the case of osteomyelitis, key SNPs within genes such as *IL-1β* (including rs1143634 and rs16944), *IL-6* (e.g., rs1800795), *VDR*, and *TNF-α* are instrumental in evaluating susceptibility. Similarly, for PJI, significant SNPs encompass those located in the *MBL* gene (-550 G/C, -54 G/A), the *IL-1β* gene (rs16944), and specific polymorphisms of the *IL-6* gene. By integrating genetic screening into clinical protocols, healthcare professionals can more effectively identify individuals with an elevated risk, customize personalized treatment plans, and administer targeted preventive strategies, thereby enhancing patient outcomes and mitigating the occurrence of these infections.

### 4.2 Personalized treatment

The comprehension of the functional role of specific SNPs in osteomyelitis and PJI serves as a valuable tool in the development of tailored treatment strategies, which ultimately enhances therapeutic efficacy and mitigates undesired side effects. In the case of high-risk patients harboring *IL-1* gene polymorphisms, such as rs16944, the utilization of IL-1 receptor antagonists, such as Anakinra, can significantly attenuate inflammatory responses (110). Furthermore, the identification of individuals with *TLR* gene polymorphisms may suggest an increased susceptibility to certain antibiotics, enabling the customization of antibiotic regimens to bolster effectiveness and diminish the potential for antibiotic resistance (111).

In cases where patients are identified as possessing high-risk gene polymorphisms, it is advisable to adopt more stringent monitoring and care protocols both before and after surgical procedures to mitigate the potential risk of postoperative infection (112). Prior to surgery, preparatory measures for patients with high-risk PJI profiles may entail prophylactic antibiotic therapy tailored to their specific genetic makeup, thus substantially diminishing the likelihood of infection (113). Following surgical procedures, individuals with specific SNP should undergo rigorous management strategies that incorporate close monitoring to promptly identify and address any signs of infection. This entails conducting regular blood tests, imaging assessments, and swift intervention upon the earliest manifestation of infection symptoms. The execution of these genotype-specific strategies is aimed at enhancing the quality of patient care, while also fostering a more efficient allocation of medical resources. Consequently, this approach contributes to the reduction of overall healthcare costs and the optimization of long-term outcomes for patients afflicted with osteomyelitis and PJI.

### 4.3 New drug development and therapeutic targets

Investigating the precise mechanisms of SNPs possesses considerable potential to propel the progression of novel targeted therapies, thereby providing patients with more efficacious and individualized treatment approaches. For instance, the investigation of genetic variations within the *TNF-α* gene, such as the rs1799964 polymorphism, has the capability to enable the development of therapeutic strategies that specifically modulate TNF-α activity. Additionally, medications like infliximab and adalimumab, which serve as TNF-α inhibitors, have demonstrated efficacy in managing osteomyelitis and mitigating inflammatory responses (114, 115). Furthermore, for individuals who possess MBL gene polymorphisms that lead to decreased MBL levels, the development of MBL replacement therapies holds the potential to fortify immune responses, consequently mitigating the risk of infection and optimizing overall patient wellbeing (116, 117).

Similarly, the application of precise inhibitors that specifically target genetic variations in the *IL-6* gene, notably the rs1800795 polymorphism, effectively reduces inflammation and arrests the progression of osteomyelitis and PJI. Medications, such as tocilizumab and sarilumab, which function as antagonists to the IL-6 receptor, have exhibited substantial therapeutic efficacy in these scenarios (118, 119). By leveraging these genetic insights, healthcare providers are capable of developing highly personalized therapeutic strategies that target the underlying genetic factors of these conditions, thereby enhancing the effectiveness and individualization of patient care.

Genetic polymorphism data can be leveraged to precisely calibrate drug dosages, thereby optimizing therapeutic efficacy while minimizing the occurrence of undesirable side effects (120, 121). Specifically, the adjustment of anti-inflammatory medication dosages in accordance with the genetic variations of *TNF-α* and *IL-6* genes ensures that patients receive individualized, optimal, and effective treatments that are specifically tailored to their unique genetic profiles (122, 123). This tailored strategy significantly improves the efficacy of the treatment while markedly reducing the potential for adverse reactions and complications.

### 4.4 Improved prevention strategies

The identification of individuals harboring high-risk gene polymorphisms enables the implementation of comprehensive preventative measures in both lifestyle adjustments and medical oversight, thereby minimizing the likelihood of disease occurrence (124). It is imperative to provide focused health education to these individuals, highlighting the significance of maintaining optimal hygiene, avoiding traumatic injuries, and adhering to recommended wound care practices (125). Furthermore, the introduction of regular health screenings for high-risk populations serves to facilitate the timely detection and prompt management of potential infections (126). These screenings may encompass routine blood testing, imaging studies, and physical examinations, aimed at detecting early indicators of osteomyelitis or PJI (127, 128). Genetic risk factors-guided early intervention strategies can significantly enhance health outcomes by halting the progression of these conditions and minimizing the occurrence of severe complications.

SNPs occupy a pivotal role in the diagnosis, therapeutic intervention, and preventative measures pertaining to osteomyelitis and PJI. Genetic testing presents an opportunity for the implementation of tailored risk assessment and individualized treatment approaches, which subsequently enhance treatment effectiveness and minimize the incidence of complications. Further advancements in research endeavors and clinical applications possess the potential to refine these strategies and improve patient prognosis.

## 5 Conclusion

**Figure 1:**
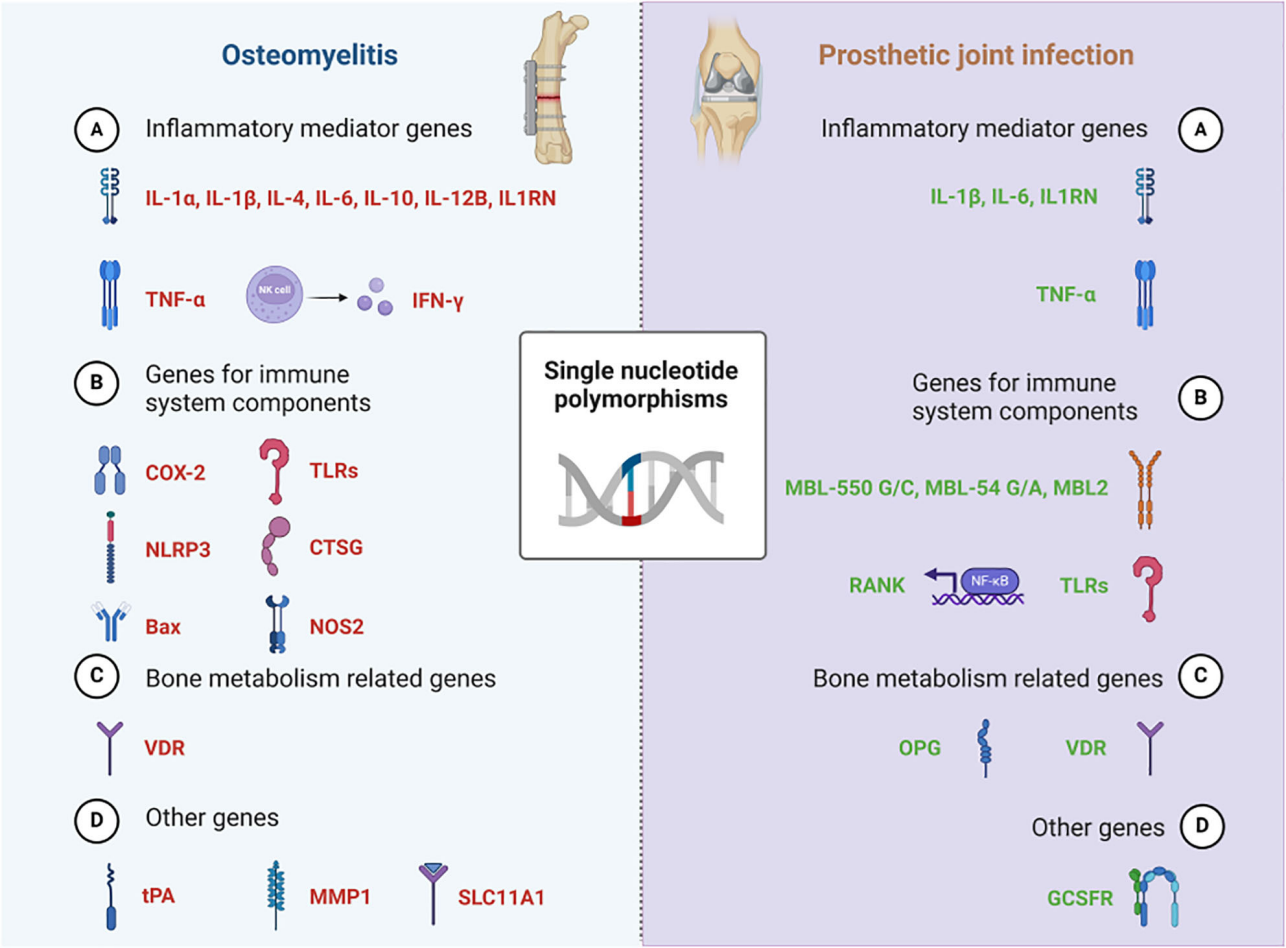
SNPs involved in Osteomyelitis & Prosthetic joint infection.

In summary, SNPs located in the genes of *IL-1α* (rs1800587), *IL-1β* (rs16944, rs1143627, rs1143634), *IL1RN* (rs2234663, rs4251961), *IL-4* (rs2243248, rs2243250, rs2070874), *IL-6* (rs1800795, rs1800796), *IL-10* (rs1800871), *IL-12B* (rs3212227), *TLR2* (rs3804099), *TNF-α* (rs1799964), *tPA* (rs4646972), *CTSG* (rs45567233), *COX-2* (rs689466), *VDR* (rs731236, rs2228570, rs7975232, rs1544410), *MMP1* (rs1799750, rs1144393), *NLRP3* (rs10754558, rs7525979) and *Bax* (G (-248) A) may play an important role in the development of osteomyelitis. SNPs located in the genes of *IL-1β* (rs16944, rs1143634), *IL1RN* (rs2234663), *IL-6* (-174 G/C, A/G nt565), *TNF-α* (rs361525), *MBL-550 G/C*, *MBL-54 G/A*, *MBL2* (rs11003125), *OPG* (rs3102735), *RANK + 575 C/T*, *VDR-T*, *GCSFR* may be involved in the PJI (**Figure 1**, **Table 1**).

## 6 Limitations and future perspectives

**Figure 2:**
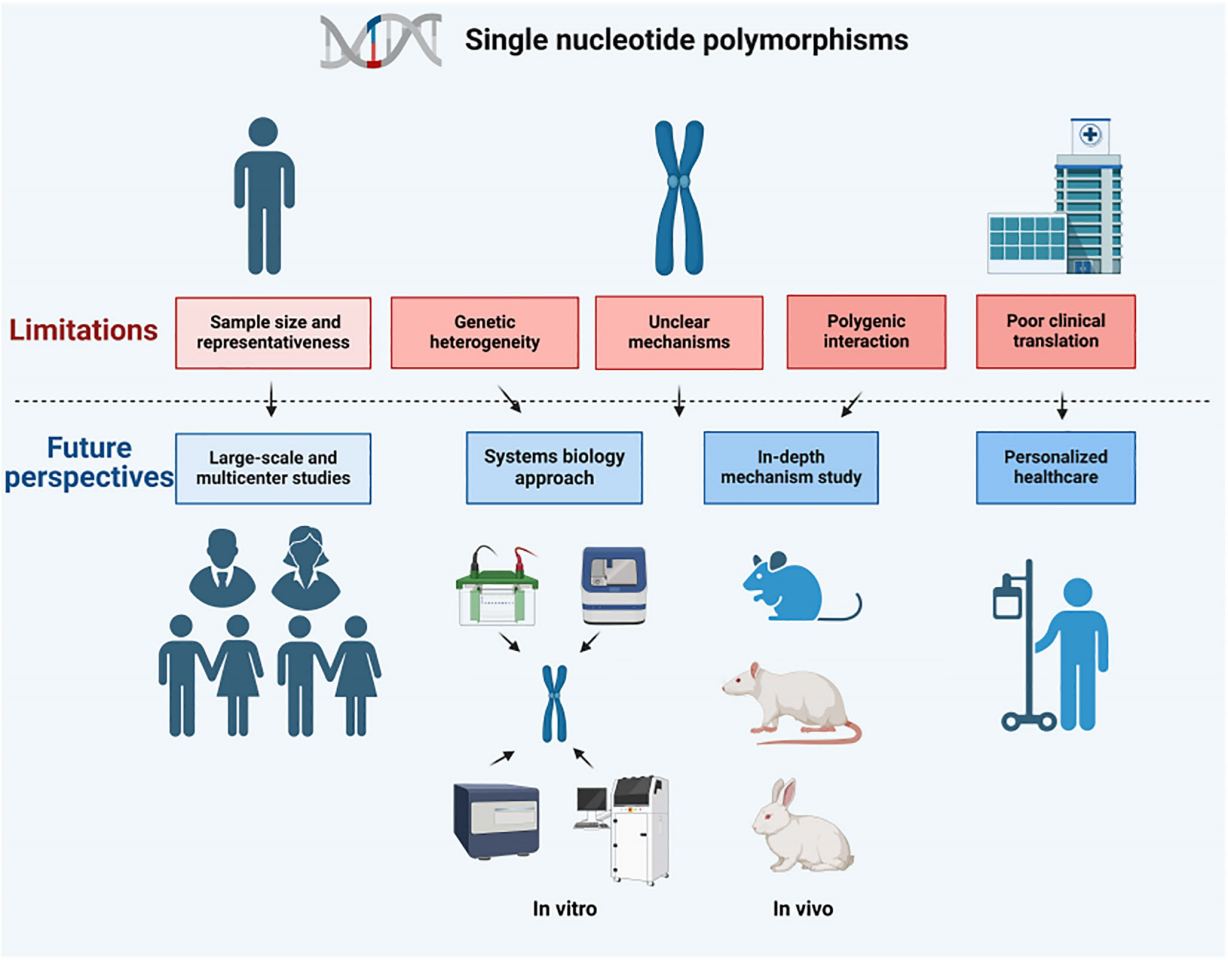
Limitations and future perspectives for SNPs.

In the realm of research pertaining to osteomyelitis and PJI, SNPs have yielded pivotal insights into the intricate interplay between genetic predisposition and the susceptibility to, as well as the progression of, these diseases. Despite remarkable strides made in this field, the investigation of SNPs remains confronted with a multitude of constraints and hurdles within the clinical arena. The subsequent paragraphs delve into these limitations in a comprehensive manner, and further propose avenues for future exploration (**Figure 2**).

### 6.1 Limitations

(1) Numerous investigations into the relationship between SNPs and osteomyelitis or PJI have been undertaken, relying predominantly on small sample sizes. This approach, however, carries the risk of introducing statistical bias and confines the applicability of the findings. Furthermore, these studies tend to concentrate on specific racial or geographical cohorts, neglecting a holistic, multiracial, and multiregional perspective. The majority of rigorous and methodical research endeavors exploring the correlation between SNPs and both osteomyelitis and PJI have primarily aimed to illuminate the intricate interplay between genetic variations and the predispositions to these diseases. These studies generally explore the potential connections between various SNP genotypes and the probability of disease development, while limited efforts have been directed towards an exhaustive evaluation of sample characteristics, considering the differing prevalence rates among distinct SNP genotypes. (2) A discernible gap is evident in the realm of subgroup analyses, where only a limited number of studies have briefly acknowledged patient age and other relevant susceptibility factors. However, these variables have rarely been subjected to rigorous examination. In particular, the systematic investigation of the characteristic patterns of age groups under varying SNP genotypes and their subsequent impacts on disease progression remains scarce. Consequently, this limitation restricts the breadth and depth of research endeavors aimed at elucidating the influence of diverse genotypes on disease predisposition. (3) Another significant constraint lies in the inherent limitations of the primary data. Studies that have undertaken direct comparisons of the occurrences of osteomyelitis and PJI across various SNP genotypes have frequently refrained from divulging comprehensive patient information. This lack of detailed data has, as a result, obstructed further statistical investigations, thereby restricting our capacity to delve deeply into the specific pathological characteristics and their contributory factors within the framework of distinct SNP genotypes. (4) Even for individuals with the same disease, their genetic backgrounds exhibit significant variations (129–131). Given the genetic heterogeneity, it becomes challenging to discern the distinct impacts of individual SNPs amidst other confounding genetic and environmental factors (132). (5) Despite the identification of numerous SNPs that are linked to osteomyelitis and PJI, the precise biological pathways through which these SNPs influence disease progression remain elusive. The absence of thorough mechanistic investigations hinders the effective translation of this genetic knowledge into clinical practice. (6) Multi-gene interactions play a pivotal role in the development of osteomyelitis and PJI, which are typically not attributed to a solitary genetic variant, but rather to a complex interplay of multiple genes (133, 134). Current research often ignores the complexity of this genetic network. (7) Despite the identification of correlations between genes and diseases, there persist formidable challenges in converting this knowledge into viable diagnostic and therapeutic methodologies (135, 136), especially in genetic counseling, risk assessment, and treatment decisions (137).

### 6.2 Future perspectives

(1) Large-scale and multi-center studies: The representativeness of the study and the reliability of the results can be enhanced by expanding the sample size and covering more races and regions. Such studies can help define more precisely the impacts of SNPs on disease susceptibilities. Furthermore, researchers are strongly encouraged to provide comprehensive raw data, thereby enabling the scientific community to conduct further analysis and validation, ultimately leading to a deeper understanding of the intricate relationships between SNPs and disease susceptibilities.

(2) Systems biology approach: Adopting a rigorous systems biology methodology to investigate the intricate interplay between multiple SNPs and other genetic elements within a network framework can potentially uncover the complex underlying genetic architecture of diseases. Furthermore, the implementation of sophisticated subgroup analysis methodologies is intended to clarify the impacts of age, pathological conditions, and other pertinent variables on disease predisposition among individuals with diverse SNP genotypes.

(3) In-depth mechanistic studies: To investigate the impact of particular SNPs on the progression of diseases, research endeavors should delve into their mechanisms of modulating cell signaling, governing gene expression regulation, and influencing other biological pathways. This can be achieved through rigorous experimentation utilizing *in vitro* systems and animal models, ensuring a comprehensive and scientific approach to elucidating their role in disease development.

(4) Preclinical and translational research: To advance the field of preclinical and translational research, it is essential to intensify efforts in exploring individualized preventive and therapeutic strategies that are grounded in SNPs. This includes the development of tailored drug therapies and the construction of predictive models, both of which hold the potential to significantly enhance the precision and effectiveness of medical interventions.

(5) Interdisciplinary collaboration: It is advocated for the advancement of genetic research pertaining to osteomyelitis and PJI through the concerted efforts and integration of expertise from various disciplines, such as genetics, immunology, microbiology, and clinical medicine.
